# Unusual properties and potential applications of strain BN-MS_2_ (M = Mo, W) heterostructures

**DOI:** 10.1038/s41598-019-39970-0

**Published:** 2019-03-05

**Authors:** Jie Su, Jian He, Junjing Zhang, Zhenhua Lin, Jingjing Chang, Jincheng Zhang, Yue Hao

**Affiliations:** China State Key Discipline Laboratory of Wide Band Gap Semiconductor Tecchnology, Shaanxi Joint Key Laboratory of Graphene, Advanced Interdisciplinary Research Center for Flexible Electronics, School of Microelectronics, Xidian University, Xi’an, 710071 China

## Abstract

Heterostructures receive intensive attentions due to their excellent intrinsic properties and wide applications. Here, we investigate the natural physical properties and performances of strain BN-MS_2_ (M = Mo, W) heterostructure by density functional theory. Different to compressive monolayer MS_2_, corresponding BN-MS_2_ heterostructures keep direct band-gap characters because effects of charge transfer on anti-bonding dz^2^ orbitals are stronger than those of Poisson effect. Mexican-hat-like bands without magnetic moments are observed at strain BN-MS_2_ heterostructures when the compression is enough. Consequently, electron mobilities of strain BN-MS_2_ heterostructures are slightly reduced at first and then enlarged with increasing compressive strain. Note that, strain BN-MS_2_ heterostructures reduce the band edges of MS_2_ layers and extend their application in photocatalytic water splitting. But just the n-type and p-type Schottky barriers of devices with strain BN-MS_2_ heterostructures are reduced and even vanished with the increasing tensile and compressive, respectively. Besides, electron mobilities of strain BN-MoS_2_ and BN-WS_2_ heterostructures can be enhanced to 1290 and 1926 cm^2^  V ^−1^ s^−1^, respectively, with increasing tensile strain. Interestingly, the exciton binding energies of strain BN-MS_2_ heterostructures exhibit oscillation variations, different to those of strain monolayer MS_2_.

## Introduction

Two-dimensional transition metal dichalcogenides (TMDs) are an emerging class of materials with atomic thickness, pristine surface, unique and tunable electronic properties which make them highly attractive for applications ranging from nanoelectronics to optoelectronics with high performances^1,2^. However, many studies have revealed that the performances of nanodevices based on TMDs in experiment are lower than the theoretical expectations^1–4^. For examples, most measured carrier mobilities of monolayer MoS_2_ nanodevices under room temperature are far lower than the theoretical predication of 410 cm^2^ V^−1^ s^−1^ ^5–7^. That can be ascribed to three main reasons: first, the fabricated TMDs flakes in experiment containing several defects, like vacancies, which enhance the scattering effects and deteriorate the intrinsic properties of TMDs^8,9^. Second, oxide substrates with surface roughness induce strong interfacial charged impurities at substrates-TMDs interfaces^10–14^. Third, metal-TMDs interfaces usually have large contact resistances and Schottky barrier heights (SBHs) which limit the carrier injection efficiency^15–19^. To overcome these issues, van der Waals heterostructures engineering, especially BN-TMDs heterostructures, have been employed. Moreover, such approaches have evidently improved the performances of nanodevices based on TMDs. For examples, Wang, *et al*. have fabricated MoS_2_, MoSe_2_ and WS_2_ layers on hexagonal boron nitride (BN) substrates to form BN-TMDs heterostructures, which enhanced the photoluminescence and room-temperature mobilities of TMDs due to the reduced substrate traps and improved fake quality^20–24^. Liao *et al*. have found that forming BN-TMDs heterostructures in metal-TMDs interface regions not only modulated the work function and fermi level pinning effect, but also reduced the SBHs and contacts resistances of metal-TMDs interfaces by an order of magnitude^25,26^. Moreover, BN-TMDs heterostructures between gate and channel layer could change the main noise source in channel from charged impurities to trapping-detrapping process^27^. Thus, BN-TMDs heterostructures have been widely used in the high performance nanodevices, including integration^28^, photoresponse^29^, self-biased diode^30^, etc.

It should be noted that MS_2_ (M = Mo, W) as typical members of TMDs are not only suitable for above mentioned nanodevices, but also have great potentials in applications of flexible nanodevices, such as flexible battery^31^, humidity sensing^32^, flexible supercapacitor^33^, and so on. However, the performances of flexible nanodevices based on MS_2_, like carrier mobilities, in experiment are far lower than expectation^34–36^. Moreover, direct-to-indirect transitions occur in band gaps of monolayer MS_2_ in the flexible nanodevices^37–41^. Such characters are not conductive to realize high performance flexible nanodevices. Inspired by the excellent performance of BN-TMDs heterostructure in optoelectronics as above mentioned, forming strain BN-MS_2_ heterostructure with great potential to improve the performance flexible nanodevices. However, few studies have focused on the strain BN-MS_2_ heterostructures, although the carrier mobilities of monolayer MoS_2_ flexible transistors had been enlarged from 30 to 45 cm^2^ V^−1^ s^−1^ when monolayer MoS_2_ was substituted by strain BN-MoS_2_ heterostructure^34,42^. Moreover, so far few theoretical studies have focused on the natural physical properties of strain BN-MS_2_ heterostructures. Hence, in this work, the electronic, magnetic, transport, optical properties, and additional potential application of strain BN-MS_2_ (M = Mo, W) heterostructures were comprehensively investigated by first principles calculations.

## Results and Discussion

### Geometric structure

**Figure 1:**
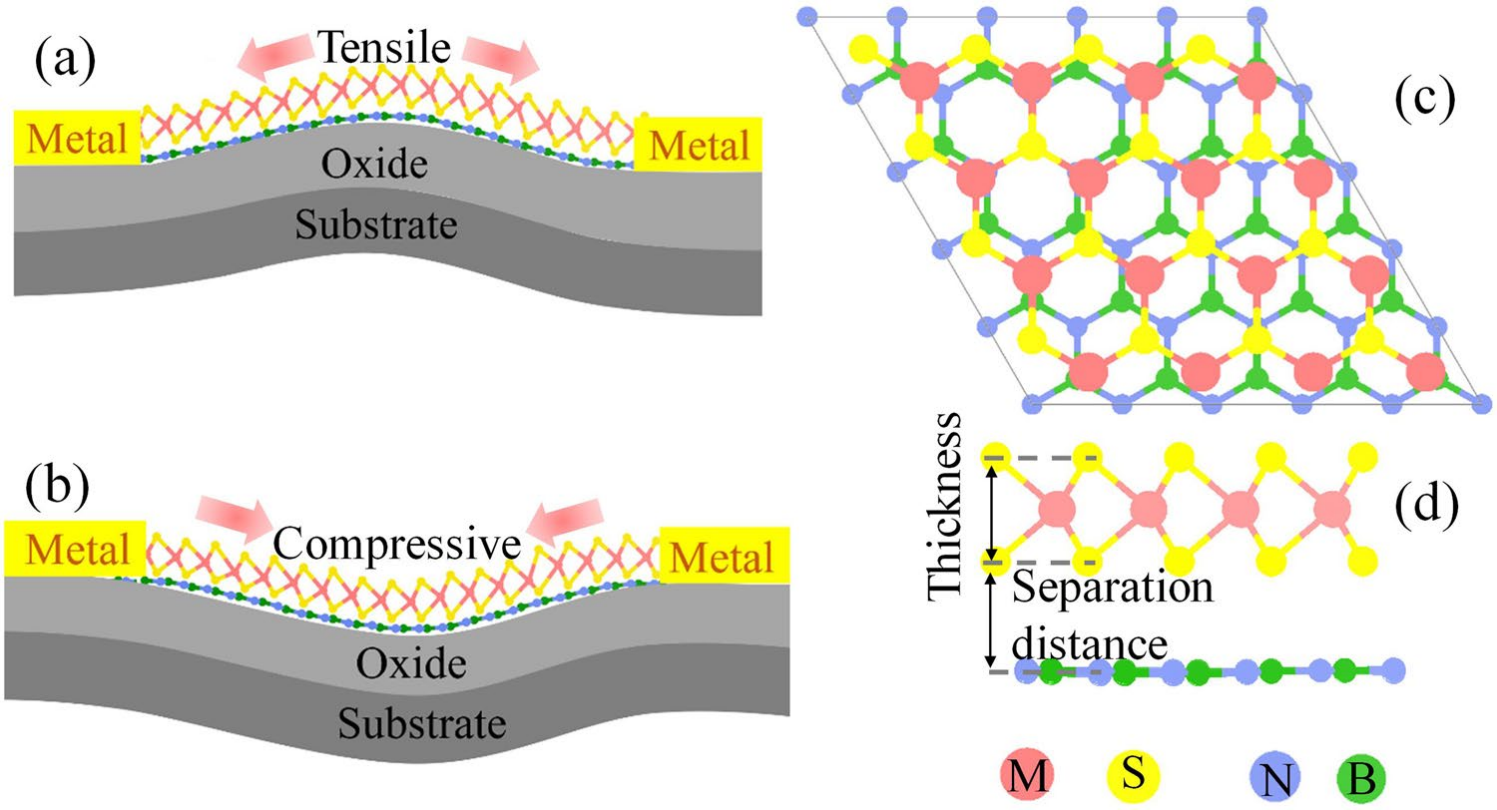
Schematics of BN-MS_2_ heterostructures nanodevices with (**a**) tensile and (**b**) compressive strain. (**c**) Top and (**d**) side views of BN-MoS_2_ heterostructures.

Before exploring the strain BN-MS_2_ (M = Mo, W) heterostructures (Fig. 1a,b), the geometric structures of isolated monolayer BN, MoS_2_, WS_2_ were investigated and listed in Table 1. The lattice constants of isolated monolayer BN and MS_2_ are in good agreement with previous studies^43–48^. BN-MS_2_ heterostructures are constructed by stacking the BN and MS_2_ monolayers on top of each other. To study the natural physical properties of strain BN-MS_2_ heterostructures accurately, 5 × 5 BN supercells are constructed and strained to match with the 4 × 4 MS_2_ supercells, as shown in Fig. 1. The lattice mismatches of BN-MoS_2_ and BN-WS_2_ heterostructures are less than 1%. The equilibrium separations of BN-MoS_2_ and BN-WS_2_ heterostructures are 3.28 Å and 3.15 Å, respectively, after extensive test.

**Table 1:** Lattice constants and band gaps of strain monolayer BN, MS_2_, and BN-MS_2_ heterostructures (M = W, Mo).

	Without strain			4% tension	4% compression
	Lattice constant (Å)	Band gap (eV)	Thickness (Å)	Thickness (Å)	Thickness (Å)
Monolayer BN	2.51, 2.49^43^, 2.50^45^	4.57, 4.82^45^	—	—	—
Monolayer MoS_2_	3.168, 3.16^47^, 3.18^48^	1.76, 1.74^47^, 1.67^48^	3.12	3.05	3.23
Monolayer WS_2_	3.153, 3.146^44^, 3.152^46^	1.91, 1.94^49^, 1.82^48^	3.15	3.07	3.25
BN-MoS_2_ heterostructure	12.672	1.72	3.13	3.05	3.22
BN-WS_2_ heterostructure	12.612	1.88	3.14	3.06	3.24

The thicknesses of isolated MS_2_ layers and MS_2_ layers in the heterostructures are also listed for comparison.

### Electronic properties

**Figure 2:**
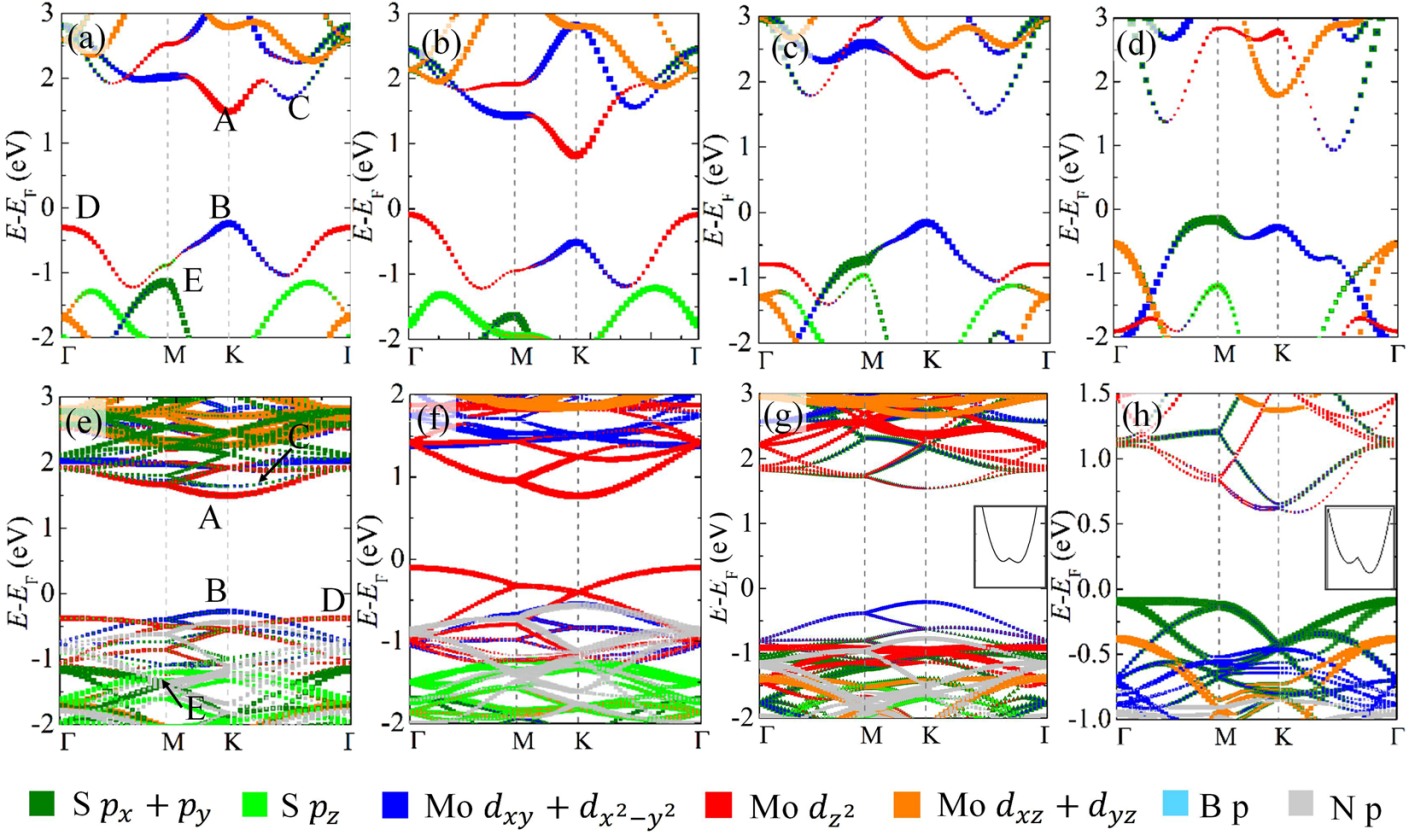
Projected band structures of monolayer MoS_2_ (**a**) without strain, and with (**b**) 4% tensile, (**c**) 4% compressive and (**d**) 10% compressive strains, respectively. Projected band structures of BN-MoS_2_ heterostructures (**e**) without strain, and with (**f**) 4% tensile, (**g**) 4% compressive and (**h**) 12% compressive strains, respectively. The insertions in (**g**) and (**h**) are the conduction bands around the K points.

To well understand the electronic properties of strain BN-MS_2_ heterostructures, the electronic structures of corresponding isolated strain monolayer MS_2_ are studied firstly. Figure 2a as an example displays the electronic structure of monolayer MoS_2_. Monolayer MoS_2_ is a direct band gap semiconductor with conduction band minimum (CBM) (*viz*. band A) and valence band maximum (VBM) (*viz*. band B) at K point. Moreover, its CBM and VBM are mainly dominated by anti-bonding ${d}_{{{\rm{z}}}^{2}}$ and bonding ${d}_{{{\rm{x}}}^{2}-{{\rm{y}}}^{2}}+{d}_{xy}$ orbitals of Mo atoms, respectively. Such characters are consistent with previous reports^41^. In addition, other special bands C, D, and E (marked in Fig. 2a) of monolayer MoS_2_ are mainly composed of anti-bonding ${d}_{{{\rm{x}}}^{2}-{{\rm{y}}}^{2}}+{d}_{xy}$ orbitals, bonding ${d}_{{{\rm{z}}}^{2}}$ orbitals of Mo atoms, and bonding ${p}_{x}+{p}_{y}$ orbitals of S atoms, respectively, as demonstrated in Fig. 2a. Similar characters, except for the band gap, are also observed in monolayer WS_2_, as shown in Fig. S1. The direct band gaps of monolayer MoS_2_ and WS_2_ are 1.76 and 1.91 eV, respectively, which are in accordance with previous reports^44–49^ and listed in Table 1. Note that, although these band gaps are lower than the experimental values^50^, they are closer to the transport band gaps in nanodevices compared to the band gap calculated by GW and HSE functional^19,51^. That is because the strong Coulombic screening by metal electrodes can minimize the exciton binding energies and many body effects of 2D materials^16,27^. Moreover, the electronic structures of MS_2_ calculated by PBE functional are similar to those calculated by HSE functional (as demonstrated in Fig. S2). It suggests that PBE functional is sufficient to study the electronic properties of MS_2_ layers and BN-MS_2_ heterostructures.

**Figure 3:**
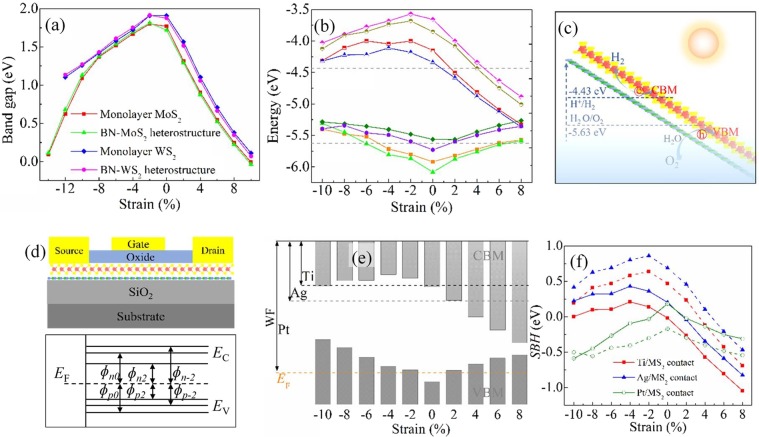
(**a**) Band gaps and (**b**) band edges of monolayer MS_2_ and BN-MS_2_ heterostructures as functions of strain, respectively. The square, triangle, rhombus, and circle indicate the monolayer MoS_2_, BN-MoS_2_ heterostructure, monolayer WS_2_, and BN-WS_2_ heterostructure, respectively. The up and low gray dash lines in Figure (**b**) represent the water reduction and oxidation potential levels, respectively. (**c**) Schematic illustration for BN-MS_2_ heterostructure used in the photocatalysis for water splitting. (**d**) Sketch of SBHs at metal/MS_2_ interfaces in transistors based on BN-MS_2_ heterostructure. (**e**) Band diagrams of metal Ti, Ag, Pt, and strain BN-MoS_2_ heterostructures. WF and *E*_F_ denote the work function and Fermi level of metal, respectively. (**f**) The ideal n-type SBHs of Ti/MS_2_ and Ag/MS_2_ contacts and p-type SBHs of Pt/MS_2_ contacts as functions of the strain BN-MS_2_ heterostructures. The solid and dash lines represent the metal/MoS_2_ and metal/WS_2_ interfaces, respectively.

When monolayer MS_2_ undergoes tensile strain, the band gap reduces gradually and accompanies with a direct-to-indirect band gap transition. That is because the thickness of MS_2_ (as listed in Table 1) is reduced by tensile strains due to the Poisson effect. Such thickness reduction strengthens the coupling between the *p*_*z*_ orbitals of S atoms and ${d}_{{{\rm{z}}}^{2}}$ orbitals of M atoms, and such biaxial tensile strain weakens the coupling between the ${p}_{x}+{p}_{y}$ orbitals of S atoms and ${d}_{{{\rm{x}}}^{2}-{{\rm{y}}}^{2}}+{d}_{xy}$ orbitals of M atoms. As a result, the energies of anti-bonding band A and bonding band B reduce, and the energies of anti-bonding band C and bonding band D enlarge. Thus, the VBM shifts up and moves from K to Γ point, and the CBM at the K point shifts down, leading to the reduction of band gaps with increasing tensile strain, as shown in Fig. 2b. On the contrary, application of compressive strain enlarges the thickness of monolayer MS_2_ (as listed in Table 1), and then weakens the coupling between the *p*_*z*_ orbitals of S atoms and ${d}_{{{\rm{z}}}^{2}}$ orbitals of M atoms and enhances coupling between the ${p}_{x}+{p}_{y}$ orbitals of S atoms and ${d}_{{{\rm{x}}}^{2}-{{\rm{y}}}^{2}}+{d}_{xy}$ orbitals of M atoms. Consequently, the anti-bonding bands A and C shift up and down, respectively. The position of CBM transforms from anti-bonding band A to anti-bonding band C, leading to a direct-to-indirect band gap transition, as displayed in Fig. 2c,d. Nevertheless, it should be noted that the energy of anti-bonding band C is higher than that of anti-bonding band A of monolayer MS_2_ without strain, and it decreases slightly with the increasing compressive strain. As a result, the energy of band C of monolayer MS_2_ with small compressive strain is still higher than that of band A of monolayer MS_2_ without strain. Thus, the band gaps of monolayer MS_2_ slightly enlarge at first and then decrease with the increasing enlarge compressive strain, as displayed in Fig. 3a, which is consistent with previous reports^41^. In addition, the enhanced coupling between the ${p}_{x}+{p}_{y}$ orbitals of S atoms and ${d}_{{{\rm{x}}}^{2}-{{\rm{y}}}^{2}}+{d}_{xy}$ orbitals of M atoms induced by compressive strain also rises the energy of bonding band E, as shown in Fig. 2c. When the compressive strain is larger than 8%, such raised bonding band E can surpass that of raised bonding band B. Consequently, the position of VBM shifts from the bonding band B at the K point to bonding band E at the M point, leading to a direct-to-indirect transition, as shown in Fig. 2d.

For BN-MS_2_ heterostructures, their CBM and VBM are dominated by the MS_2_ layer, such as the BN-MoS_2_ heterostructure in Fig. 2e. Moreover, the components and positions of CBM and VBM of BN-MS_2_ heterostructures are similar to those of monolayer MS_2_. It suggests that the intrinsic electronic properties can be remained when monolayer MS_2_ transforms to type-I BN-MS_2_ heterostructure. However, the band gaps reduce slightly to be 1.72 and 1.88 eV for BN-MoS_2_ and BN-WS_2_ heterostructures, respectively. That is because the weak charge transfer between BN and MS_2_ layers (as shown in Fig. S3) slightly reduce the anti-bonding band A. When BN-MS_2_ heterostructures undergo tensile strain, they keep type-I heterostructure characters, and their CBM and VBM are still dominated by MS_2_ layer. Moreover, variations of band gaps and band edges of tensile BN-MS_2_ heterostructures (see Fig. 2f) are similar to those of tensile monolayer MS_2_ since the biaxial tensile also induces the reduction of MS_2_ layer thickness in BN-MS_2_ heterostructures (as listed in Table 1). However, the reduction indirect band gaps of tensile BN-MS_2_ heterostructures are slightly lower than those of corresponding tensile monolayer MS_2_, as exhibited in Fig. 3a. That is because additional charges are accumulated at the MS_2_ layer of BN-MS_2_ heterostructures (as displayed in Fig. S3), which can further reduce the energy of anti-bonding ${d}_{{{\rm{z}}}^{2}}$ (*viz*. band A). In the case of BN-MS_2_ heterostructures with compressive strain, they keep type-I heterostructure characters. The MS_2_ layer of compressive BN-MS_2_ heterostructures are still direct band semiconductor with CBM and VBM located at K point, as displayed in Fig. 2g. This is different to those of corresponding monolayer MS_2_ although compressive strain also induces the enlarged thickness of MS_2_ layer. That is because although the Poisson effect rises the anti-bonding orbitals ${d}_{{{\rm{z}}}^{2}}$ and reduces the anti-bonding orbitals ${d}_{{{\rm{x}}}^{2}-{{\rm{y}}}^{2}}+{d}_{xy}$, the charge transfer between BN and MS_2_ layers reduces the anti-bonding orbitals ${d}_{{{\rm{z}}}^{2}}$ and ${d}_{{{\rm{x}}}^{2}-{{\rm{y}}}^{2}}+{d}_{xy}$. Hence, the energy of anti-bonding band C is still higher than that of anti-bonding band A at the K point which dominates the CBM of BN-MS_2_ heterostructure. It should be noted that, except for the Poisson effect induced by compressive strain, the wrinkle phenomenon is also introduced to the MS_2_ layer of BN-MS_2_ heterostructure as the compressive strain continue to increases, which induce a heterogeneous charge transfer between BN and MS_2_ layers of BN-MS_2_ heterostructures (as displayed in Fig. S4). As a result, the energy of anti-bonding orbitals ${d}_{{{\rm{x}}}^{2}-{{\rm{y}}}^{2}}+{d}_{xy}$ is close to that of anti-bonding orbitals ${d}_{{{\rm{z}}}^{2}}$, and a Mexican-hat-like band around K point is formed, as shown in Fig. 2g,h. In general, the Mexican-hat-bands suggesting an obviously magnetic moments^52^. Nevertheless, nonmagnetic states are observed for BN-TMDs with larger compressive strain, as displayed in Fig. S5. When the compressive strain continues enlarging, such a Mexican-hat-like band becomes more evident, and a direct-to-indirect band gap transition is observed, as displayed in Fig. 2h. That is because the weak interaction is insufficient to hamper the increasing energy of the bonding band E at the M point.

### Band levels and applications

Figure 3b displays the band edges of BN-MS_2_ heterostructures and monolayer MS_2_ on an absolute energy scale with respect to the vacuum level. For monolayer MS_2_, their band edges of VBM and CBM states of monolayer MoS_2_ and WS_2_ are −5.97, −4.18 eV and, −5.56, −3.63 eV, respectively, which are consistent with previous reports^49^. The VBMs are enhanced with both the increasing compressive and tensile strains since compressive and tensile strain can enhance the bonding band B and bonding band D, respectively. The CBMs reduce monotonously with the increasing tensile strain, while they enlarge at first and then reduce with the increasing compressive strain due to the transfer of CBM due to the transformation of CBM, as above analysis. For strain BN-MS_2_ heterostructures, similar characters are observed. Nevertheless, the band edges of BN-MS_2_ heterostructures are lower than those of corresponding MS_2_ due to the charge transfer between BN and MS_2_ layer, as above analysis.

It is well known that the band levels are related to the applications in photocatalytic and electronic fields. Figure 3c demonstrates the schematic of photocatalytic water splitting. A good photocatalytic material for water-splitting requires that the CBM and VBM are lower and higher than the reduction and oxidation potentials of water, respectively^49^. Thus, monolayer WS_2_ is not a good photocatalytic material, as displayed in Fig. 3b. However, the CBM and VBM of BN-WS_2_ heterostructures are higher and lower than reduction and oxidation potentials of water, respectively. Moreover, such characteristics remain when the compressive and tensile strains of BN-WS_2_ heterostructures are lower than 2%. In other words, forming BN-WS_2_ heterostructures can extend the application of monolayer WS_2_ in the photocatalytic water splitting. For the monolayer MoS_2_, its band edges are outside of the reduction and oxidation potentials of water when compressive strain is lower than 4%. It means that monolayer MoS_2_ may be suitable for the application of photocatalytic water splitting, but the compressive strain is limited to be 4%. Upon forming BN-MoS_2_ heterostructure, similar characters can be observed when the compressive strain is up to 6%. It suggests that forming BN-MoS_2_ heterostructures can extend the application of strain monolayer MoS_2_ in photocatalytic water splitting.

Figure 3d displays the schematic diagrams of metal/semiconductor contacts in FETs which is usually used to calculate ideal SBH values according to Schottky-Mott rule without Fermi level pinning. For metal/MS_2_ contacts with strong Fermi level pinning effects, their large SBHs are difficult to be obtained by such methods, and they are difficult to be reduced and vanished by strained monolayer MS_2_. Different to metal/MS_2_ contacts, metal/MoS_2_ contacts with BN-MoS_2_ heterostructures show negligible Fermi level pinning effect^16,18^. The SBHs at metal/MoS_2_ contacts with strain BN-MS_2_ heterostructures can be obtained directly from the difference between band levels heterostructures and work functions (WFs) of metal electrodes. Figure 3e as an example shows the relationship between the WFs of metal and strain BN-MoS_2_ heterostructure. No matter what the work function of metal electrode, the n-type and p-type SBHs reduce with the increasing tensile and compressive strain of BN-MoS_2_ heterostructures, respectively. The detailed variations of SBHs of metal/MoS_2_ contacts induced by strained BN-MoS_2_ heterostructures as displayed in Fig. 3f. The ideal n-type SBHs of Ti/MoS_2_, and Ag/MoS_2_ contacts with BN-MoS_2_ heterostructures are −0.02 and 0.20 eV, respectively, which are lower than those of pure metal/MoS_2_ contacts^16,18^. When BN-MoS_2_ heterostructures undergo tensile strain, these n-type SBHs continue reducing and even vanishing, as exhibited in Fig. 3f. For examples, the lowest n-type SBHs of Ti/MoS_2_, and Ag/MoS_2_ contacts with tensile BN-MoS_2_ heterostructures are low to −1.04 and −0.83 eV, respectively. In addition, the p-type SBH of Pt/MoS_2_ contact with BN-MoS_2_ heterostructure is about 0.19 eV which is far lower than that of pure Pt/MoS_2_ contact^16,18^. Moreover, such p-type SBH can be further vanished when the compressive strain is larger than 2%, as shown in Fig. 3f. As to metal/WS_2_ contacts with strain BN-WS_2_ heterostructures, similar characteristics are also found, as displayed in Fig. 3f. Such results indicate that substituting strain monolayer MS_2_ in flexible devices by strain BN-MS_2_ heterostructures can realize high performance MS_2_ devices with low contact properties.

### Transport properties

**Figure 4:**
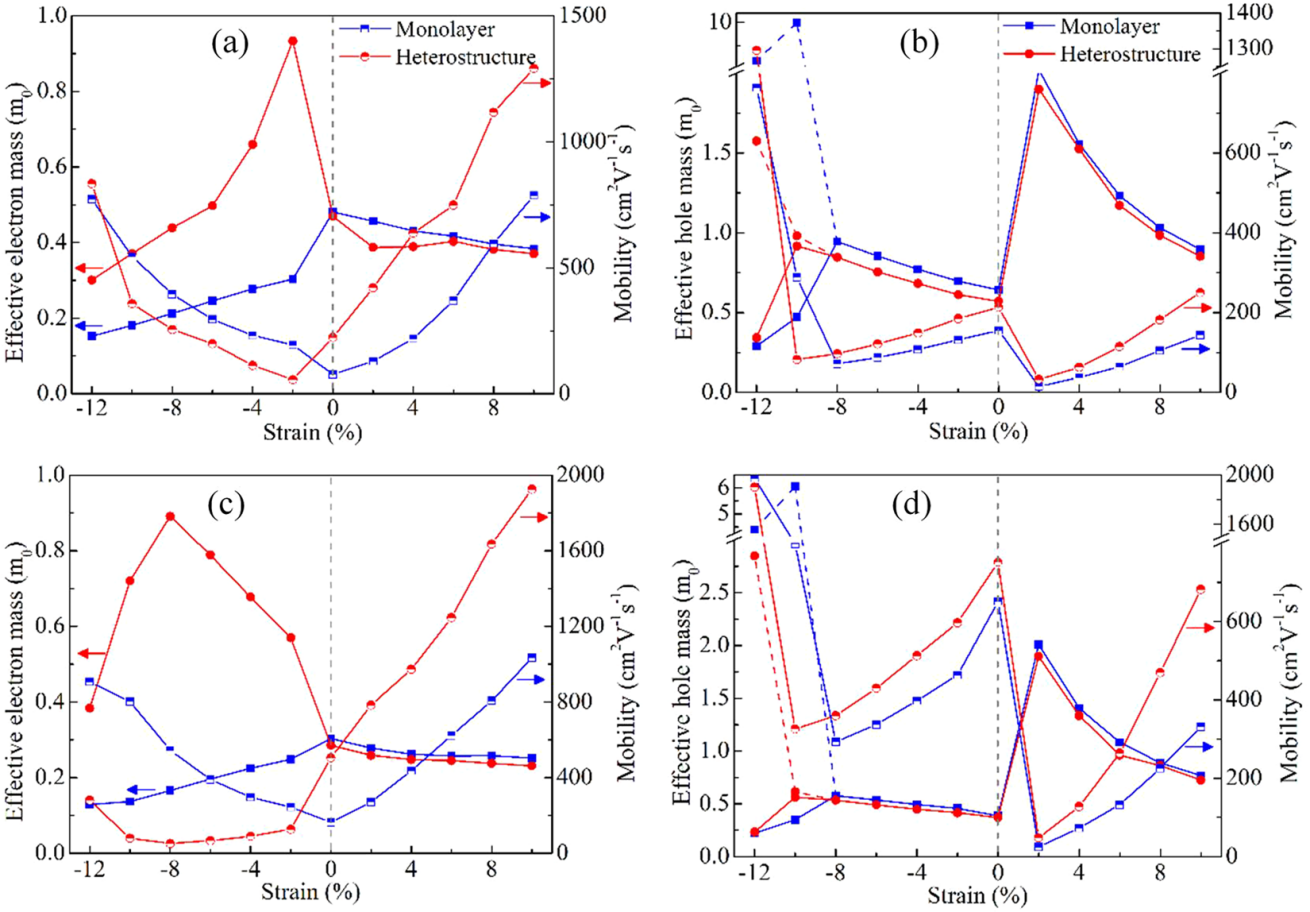
Effective masses and room-temperature mobilities of strain monolayer MS_2_ and BN-MS_2_ heterostructures: (**a**) electron of MoS_2_, (**b**) hole of MoS_2_, (**c**) electron of WS_2_, (**d**) hole of WS_2_.

Figure 4 gives the carrier effective masses and room-temperature mobilities of strained monolayer MS_2_ and BN-MS_2_ heterostructures. For monolayer MS_2_, the electron and hole mobilities at room-temperature of monolayer MoS_2_ and WS_2_ are 77.17, 155.79 cm^2^ V^−1^ s^−1^, and 163.22, 651.56 cm^2^ V^−1^ s^−1^, respectively, and the effective electron and hole masses of monolayer MoS_2_ and WS_2_ are 0.48, 0.64 m_0_, and 0.30, 0.38 m_0_, respectively, which are in good agreement with previous reports^48,53^. These effective electron masses reduce because either compressive or tensile strains are added to monolayer MS_2_. These effective hole masses enhance at first and then reduce with the increasing tensile strain since the position of VBM occurs a K-to-Γ transition; while they enlarge monotonically with the increasing compressive strain. Note that, when the compressive strain is larger than 8%, partial effective hole masses are reduced significantly, indicating an evidently anisotropic transport properties for monolayer MS_2_ with large compressive strain. That is because a K-to-M transition occurs at the position of VBM when the compressive strain is larger than 8%. In general, the small effective mass indicates the large carrier mobility. As a result, the variations of electron and hole mobilities of strain monolayer MS_2_ are opposite to these of corresponding effective masses, as exhibited in Fig. 4. In other words, both compressive and tensile strains can enlarge the electron mobilities of monolayer MS_2_. It should be noted that, nevertheless, the experimental electron mobilities of strain monolayer MS_2_ in flexible nanodevices are lower than those of monolayer MS_2_ due to the interfacial charged impurities induced by Si/SiO_2_ substrates with large surface roughness^4,34^.

For BN-MS_2_ heterostructures, the effective electron and hole masses of BN-MoS_2_ and BN-WS_2_ heterostructures are 0.47, 0.57 m_0_, and 0.28, 0.37 m_0_, respectively, which are slightly lower than those corresponding values of monolayer MS_2_. It means higher carrier mobilities for BN-MS_2_ heterostructures compared to monolayer MS_2_. The enhanced electron and hole mobilities of BN-MoS_2_ and BN-WS_2_ heterostructures are 223.50, 213.20 cm^2^ V^−1^ s^−1^, and 505.04, 751.63 cm^2^ V^−1^ s^−1^, respectively. When BN-MS_2_ heterostructures undergo tensile strain, their effective electron masses are lower than those of corresponding tensile monolayer MS_2_. An opposite phenomenon occurs for BN-MS_2_ heterostructures with compressive strain. Moreover, different to monolayer MS_2_, the effective electron masses of BN-MS_2_ heterostructures reduce monotonically with the increasing tensile strain, while they enlarge at first and then reduce with the increasing compressive strain since the increased compressive strain induces the formation of a Mexican-hat-like band gradually. As a result, the enhanced room-temperature electron mobilities of BN-MS_2_ heterostructures can be further enlarged with the increasing tensile strain, and are higher than those of corresponding monolayer MS_2_; while they reduce at first and then enlarge with the increasing compressive strain. It should be noted that the lowest electron mobilities of BN-MS_2_ heterostructure are close to that of monolayer MS_2_, although the electron mobilities of compressive BN-MS_2_ heterostructure are lower than those of corresponding compressive MS_2_, as shown in Fig. 4a,c. For examples, the lowest electron mobility of BN-MoS_2_ heterostructure of about 56.48 cm^2^ V^−1^ s^−1^ is close to that of monolayer MS_2_ of about 77.16 cm^2^ V^−1^ s^−1^. In addition, BN layer without dangling bonds can strongly weaken the interfacial charged scattering. Such characters suggest that flexible nanodevices based on BN-MS_2_ heterostructures can obtain higher electron mobilities than MS_2_ flexible nanodevices in experiment^42^. For the hole transport properties of BN-MS_2_ heterostructures, the variations of effective masses and mobilities are similar to those of monolayer MS_2_, except for the lower effective hole masses and higher room-temperature hole mobilities. However, the hole mobilities of BN-MS_2_ heterostructures with small tensile strain are lower than those of pure monolayer MS_2_. For example, the hole mobility of BN-MoS_2_ heterostructure with 2% tensile strain is about 32.72 cm^2^ V^−1^ s^−1^ which is far lower than that of pure monolayer MoS_2_ of about 155.79 cm^2^ V^−1^ s^−1^.

### Optical properties

**Figure 5:**
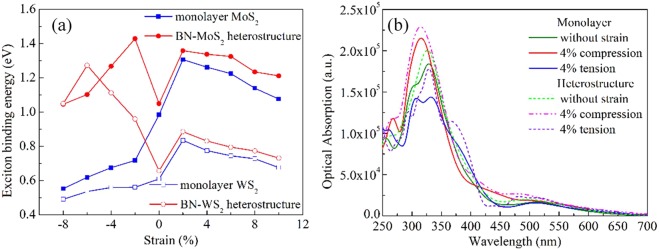
(**a**) Exciton binding energies of monolayer MS_2_ and BN-MS_2_ heterostructures as functions of strain. (**b**) Optical absorption of monolayer MoS_2_ and BN-MoS_2_ heterostructures with different strains.

Except for the transport properties, the variations of effective masses can also modulate the exciton binding energies. The calculated exciton binding energies of monolayer MoS_2_ and WS_2_ are about 0.98 and 0.61 eV, respectively, which are close to previous studies^53–58^. Moreover, these exciton binding energies enlarge at first and then reduce as the strain changes from compressive to tensile. Upon forming the BN-MoS_2_ and BN-WS_2_ heterostructures, the exciton binding energies are enhanced 1.05 and 0.65 eV, respectively, because MS_2_ layers accept charges from BN layers^55^, as shown in Fig. S3. Interestingly, the exciton binding energies of strain BN-MS_2_ heterostructures exhibit oscillation variation, just like a “M”, as demonstrated in Fig. 5a. In addition, the absorption coefficients of strain BN-MS_2_ heterostructures are enlarged compared to those monolayer MS_2_, no matter what strains are added to monolayer and BN-MS_2_ heterostructures, as the examples illustrated in Fig. 5b.

## Conclusion

In summary, electronic and optical properties of strain monolayer MS_2_ and BN-MS_2_ heterostructures and their potential performance are comprehensively investigated by density functional theory. All strain BN-MS_2_ heterostructures are type-I heterostructures, irrespective of compressive and tensile strain. However, different to the indirect band gap characters of compressive monolayer MS_2_, corresponding compressive BN-MS_2_ heterostructures keep direct band gap characters because effects of charge transfer on anti-bonding d_z2_ orbitals are stronger than those of Poisson effect. Moreover, Mexican-hat-like bands without magnetic moments are observed for compressive BN-MS_2_ heterostructures due to the non-uniform charge transfer induced by wrinkle. Consequently, electron mobilities of flexible devices with BN-MS_2_ heterostructures are reduced at first and then enlarged with the increasing compressive strain, different to those of compressive monolayer MS_2_. In addition, although strain can induce similar variations of band edges between BN-MS_2_ heterostructures and monolayer MS_2_, and extend their application in photocatalytic water splitting, strain just can reduce the Schottky barriers of devices with BN-MS_2_ heterostructures. Moreover, the n-type and p-type Schottky barriers are reduced and even vanished with the increasing tensile and compressive strain, respectively. For tensile BN-MS_2_ heterostructures, variations of their transport properties are similar to those of monolayer MS_2_, except for higher electron and hole mobilities and lower effective electron and hole masses. The room-temperature electron mobilities of MoS_2_ and WS_2_ layers in tensile BN-MS_2_ heterostructures can be up to 1290 and 1926 cm^2^ V^−1^ s^−1^, respectively. In addition, the exciton binding energies of strain BN-MS_2_ heterostructures exhibit oscillation variations, different to those of strain monolayer MS_2_.

## Computational Methodology

All calculations were performed within first-principles density functional theory (DFT) using projector augmented-wave (PAW) pseudopotentials, as implemented in the Vienna Ab Initio Simulation Package (VASP)^59,60^. The Generalized Gradient Approximaton (GGA) parameterized by Perdew-Burke-Ernzerhof (PBE)^61^ was employed to adopt for the exchange-correction functional. The van der Waals (vdW) interactions were considered using the method of Grimme (D2). The cut-off energy was set to be 450 eV. The convergence criterions were 1 × 10^−6^ eV for the self-consistent field energy and 0.01 eV/Å for the residual forces on each atom, respectively. The Monkhorst-Pack k-point mesh was sampled with a separation of about 0.015 Å^−1^ in the Brillouin zone during the relaxation and electronic calculation periods. To minimize the interlayer interactions under the periodic boundary condition, vacuums of 15 Å were added perpendicular to the layer planes of heterostructure.

The carrier mobilities were calculated by the following expression^62^,

$$\mu =\frac{2e{{\rm{\hbar }}}^{3}C}{3{k}_{B}T{|{m}^{\ast }|}^{2}{E}^{2}}$$

where *ħ* is the Planck constant, *k*_B_ is Boltzmann constant, *T* is the temperature (set to be 300 K). m* is the effective mass which is calculated by ${m}^{\ast }={\hslash }^{2}{[{\partial }^{2} {\mathcal E} (k)/\partial {k}^{2}]}^{-1}$. *E* is the deformation potential constant which denotes the shift of the band edges induced by strain. *C* is the elastic modulus of a uniformly deformed crystal for simulating the lattice distortion activated by the strain, defined by *C* = [*∂*^2^*E*/*∂δ*^2^]/*S*_0_, where *E* is the total energy of the supercell, *δ* is the applied uniaxial strain, and *S*_0_ is the area of the optimized supercell.

All the exciton binding energies of monolayer MS_2_ and BN-MS_2_ heterostructures were calculated by adopting the simplified hydrogen-like Wannier-Mott exciton modes^56,57^

$${E}_{b}={\mu }_{ex}{R}_{y}/{m}_{0}{\varepsilon }_{r}^{2}$$

where *E*_b_ is the excition binding energy, *μ*_ex_ is the reduced exciton mass (*μ*_ex_ = *m*_e_ × *m*_h_/(*m*_e_ + *m*_h_)), *m*_e_ and *m*_h_ are the effective electron and hole masses, respectively, *R*_y_ is the atomic Rydberg energy, and ε_r_ is the relative dielectric constant.

## Supplementary information

supporting information
