# Supplementary material for: Unusual properties and potential applications of strain BN-MS2 (M = Mo, W) heterostructures
Source: Sci Rep. 2019 Mar 5;9:3518. doi: 10.1038/s41598-019-39970-0 (PMC6401128; doi:10.1038/s41598-019-39970-0)
Supplement: Supplementary file 1 — supporting information [file 41598_2019_39970_MOESM1_ESM.docx]

**Supporting Information**

**Unusual properties and potential applications of strain BN-MS_2_ (M=Mo, W) heterostructures**

Jie Su^†^, Jian He^†^, Junjing Zhang^†^, Zhenhua Lin^†^, Jingjing Chang^†*^, Jincheng Zhang^†^, Hao Yue^†^

^†^China State Key Discipline Laboratory of Wide Band Gap Semiconductor Tecchnology, Shaanxi Joint Key Laboratory of Graphene, Advanced Interdisciplinary Research Center for Flexible Electronics, School of Microelectronics, Xidian University, Xi’an, 710071, China.

E-mail: jjingchang@xidian.edu.cn


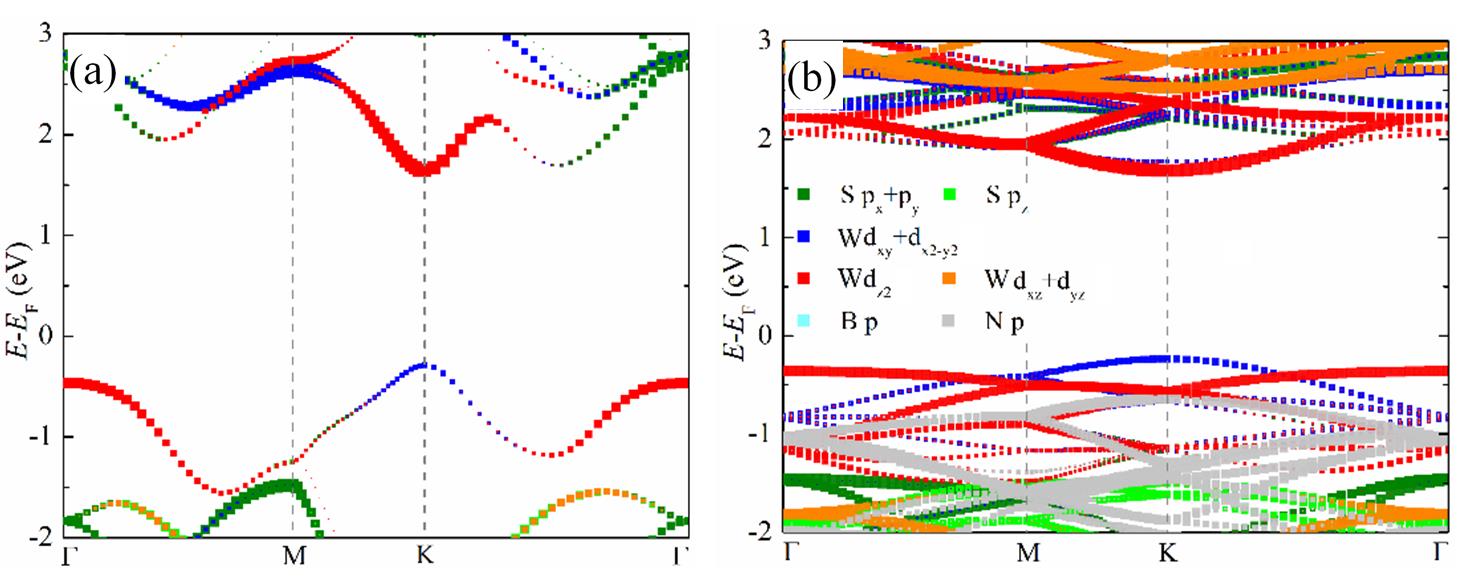


Figure S1. Projected band structures of (a) monolayer WS_2_ and (b) BN-WS_2_ heterostructure, respectively.


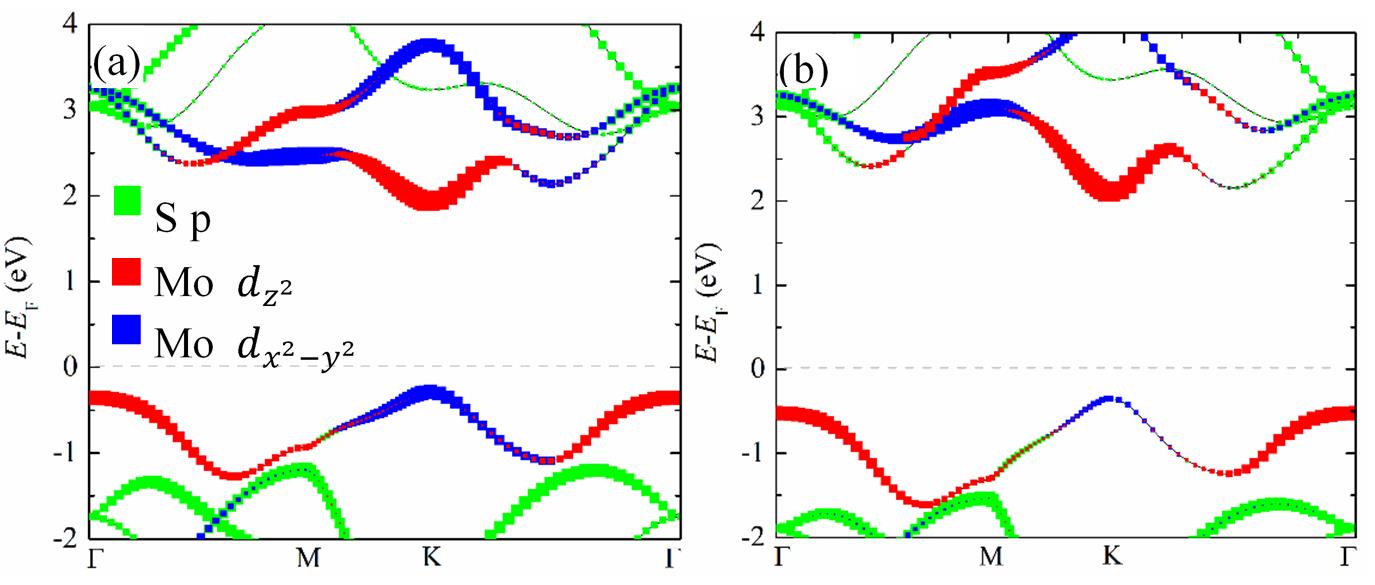


Figure S2. Projected band structures of (a) monolayer MoS_2_ and (b) WS_2_ calculated by HSE functional, respectively.


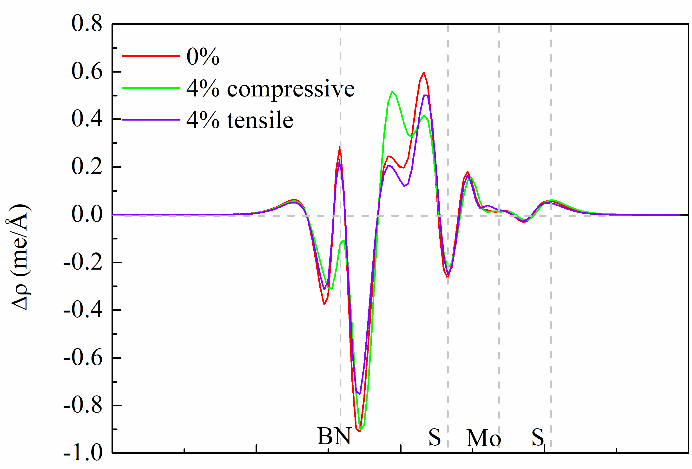


Figure S3. Plane-averaged charge density difference along the direction vertical to the BN-MS_2_ heterostructure. Positive region represents the charge accumulation, negative region represents the charge depletion.


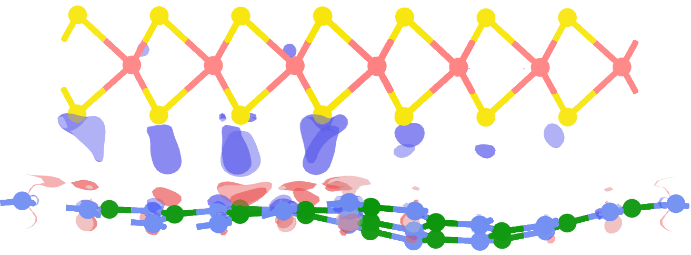


Figure S4. The 3D charge density difference of BN-MoS_2_ heterostructure with 4% compressive strain. The blue and red colors correspond to the charge accumulation and depletion, respectively.


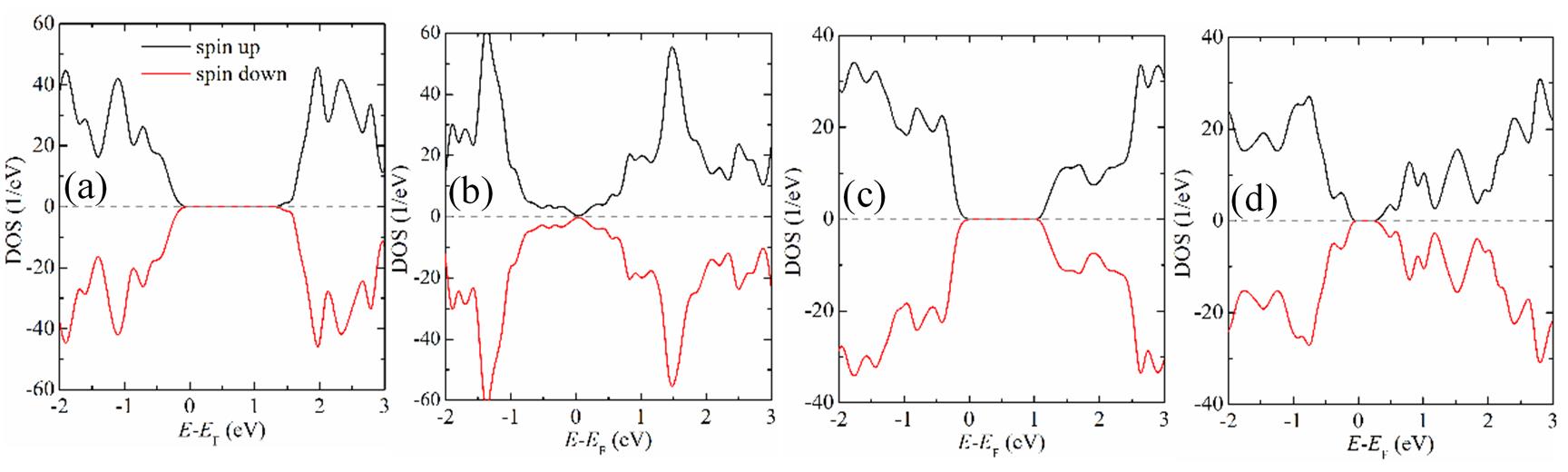


Figure S5. Spin density of states of BN-MoS_2_ heterostructures without strain (a) and with 8% tensile (b), 8% compressive (c), and 12% compressive strains (d), respectively.
